# The influence of physical activity, social relationships, and diet intake on depression: a case-series study

**DOI:** 10.1097/MS9.0000000000000406

**Published:** 2023-04-05

**Authors:** Samira Nabdi, Said Boujraf, Mohammed Benzagmout

**Affiliations:** a Faculty of Medicine and Pharmacy of Fez, Sidi Mohamed Ben Abdellah University; b Clinical Neuroscience Laboratory, Faculty of Medicine, Sidi Mohamed Ben Abdellah University; c Department of Neurosurgery, Hassan II University Hospital, Fez, Morocco

**Keywords:** depression, Mediterranean diet, physical activity, social relationships

## Abstract

This paper aims to investigate the three areas [diet, physical activity (PA), and social relationship] and their relationship with depressive disorders in the North African population.

**Study design:**

This is an observational cross-sectional study of 654 participants residing in the urban commune of Fez (*n*=326) and the rural commune of Loulja (*n*=328) in the province of Taounate. Participants were categorized into two groups: G1 without a current depressive episode and G2 with a current depressive episode. Risk factors, including locality, gender, marital status, age, parental status, employment status, tobacco use, alcohol consumption, social habits, and dietary patterns, were assessed. A multinomial probit model in Stata software was used to identify factors associated with depression occurrence in the population.

**Results:**

A total of 94.52% of the participants who engaged in PA did not experience a depressive episode (*P*=0.001). Additionally, 45.39% of the participants in our series were on a processed diet and presented with a depressive disorder (*P*=0.0001), the social contact (time spent with friends >1.5 h) remained strongly associated with reduced depressive symptoms when comparing the two groups (*P*=0.001). The results showed that being rural, a smoker, an alcohol user, and having no spouse significantly increased depression in participants. The coefficient of age was negatively related to the probability of the occurrence of age-related depression; however, this factor was not significant in the model. Thus, having a spouse and/or children and spending time with friends on a healthy diet significantly decreased depression in our population.

**Discussion:**

The converging evidence suggests that physical exercise, a stable social relationship, a healthy diet, and the use of PA can alleviate depression symptoms, but limited understanding and few studies have attempted to characterize or identify the neural mechanisms of these effects.

**Conclusion:**

Nonpharmaceutical interventions such as PA and dietary changes have proven to be effective treatments for depression, while maintaining positive social relationships can act as a protective factor, serving a prophylactic role in the prevention of depression.

## Introduction

HIGHLIGHTS

- Physical activity and social relationships are nonpharmaceutical treatments of choice for depression.
- There is a significant inverse association between diet quality and depression and anxiety.
- Being rural, a smoker, an alcohol user, and having no spouse significantly increased depression.

Major depressive disorder is a serious mental disorder that has a significant impact on an individual’s overall well-being. It is a leading cause of disability worldwide, with ∼350 million people currently affected by this condition^1^. While the underlying causes of major depressive disorder are not well understood, recent research has focused on the role of psychological stress in its development^1^–^3^.

Several factors have been identified as playing a crucial role in the development of depressive disorders, including diet, social relationships, and physical activity (PA). The relationship between diet, mood disorders, and obesity is complex and bidirectional^4^–^6^. Meanwhile, evidence suggests that physical exercise and stable social relationships can help alleviate symptoms of depression. However, few studies have attempted to characterize or identify the neural mechanisms behind these effects^2^,^7^–^9^.

The purpose of this paper is to investigate the possible role of diet, PA, and social relationships in the occurrence of depressive disorder in the population. Specifically, we aim to answer the following question: Do these three factors play a role in the development of this disorder? This case series has been reported in accordance with the PROCESS (Preferred Reporting Of CasE Series in Surgery) criteria^10^.

## Materials and methods

### Study design

The study design used in this research was a cross-sectional study. Data were collected at a single time point, and no follow-up was conducted.

### Study population

The study population comprised individuals from the North African population. A total of 654 participants were enrolled, with 326 residing in the urban commune of Fez and 328 in the rural commune of Loulja in the province of Taounate.

### Sampling technique

A nonprobability sampling technique, specifically a convenience sampling method, was used to select participants for the study. Participants were recruited from healthcare centers in the two communes.

### Inclusion criteria

To be eligible for the study, participants had to be aged 15 years and above and provide informed consent to participate in the study.

### Data collection

The study collected data using a structured questionnaire administered by trained researchers to collect information on demographics, PA, diet, social relationships, and depressive symptoms. The Mini test was used to assess depression, while PA was assessed using the IPAQ (International Physical Activity Questionnaire). Information on age, gender, socioeconomic status, meal pattern, meal composition, etc., was obtained, and a short parental questionnaire was distributed to assess individual socioeconomic status factors. Written informed consent was obtained from the participant’s parents.

### Data analysis

The data collected were analyzed using the Stata software. Categorical data were summarized as frequencies and cross-tabulations, while continuous variables were summarized as mean and variation. The *χ* ^2^ significance test was used to compare groups, with a two-sided *P* value of 0.05. A multinomial probit model was employed to determine the causal relationship between the different factors influencing depression in the population. The variables examined included locality, gender, having a spouse, age, having children, working, tobacco smoker, tobacco exposure – work, tobacco exposure – home, double tobacco exposure, alcohol consumption, time spent with friends, Mediterranean diet (MD), mixed diet, poor diet, and processed diet. To ensure a rigorous analysis, an exploitation sheet was used. The statistical significance was set at *P* less than 0.05.

## Results

### Sociodemographic profile of the population

The characteristics of the individuals are divided into groups based on the presence or absence of current depressive disorder as well as epidemiological considerations which are presented in Table 1.

**Table 1:** The characteristics of our population – current depressive episode

	Current depressive episode		
	G1 (%) – No	G2 (%) – Yes	*P*
Age group			
15–24	133 (78.7)	36 (21.3)	**0.003**
25–34	112 (66.27)	57 (33.73)	
35–49	129 (78.66)	35 (21.34)	
50–64	69 (69)	31 (31)	
≥65	46 (88.46)	6 (11.54)	
Gender			
Men	240 (74.77)	81 (25.23)	0.998
Women	249 (74.77)	84 (25.23)	
Marital status			
Single	172 (75.77)	55 (24.23)	**0.036**
Married	279 (76.02)	88 (23.98)	
Divorced	15 (62.5)	9 (37.50)	
Widower	10 (47.62)	11 (52.38)	
Remarried after divorce	11 (84.62)	2 (15.38)	
Remarried after widowhood	2 (100)	0 (0)	
The practice of a professional activity			
Yes	194 (75.45)	69 (26.24)	0.627
No	295 (75.45)	96 (24.55)	
Location			
Fez	249 (76.38)	77 (23.62)	0.345
Loulja	240 (74.77)	88 (25.23)	

Bold *P*-value of 0.003 and 0.036 suggest that the probability of obtaining the observed result by chance alone is very low.

**Figure 1:**
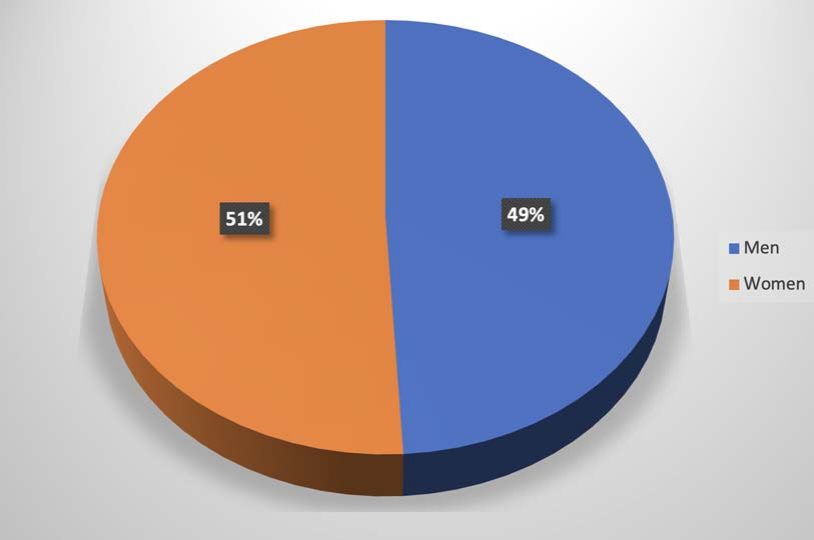
The gender distribution of the population.

Females were the majority, accounting for 50.9% of the study population, while males accounted for 49.1%, resulting in a sex ratio of 0.96 (M/F) (Fig. 1).

The mean age of the patients was 42.57±17.44 years, with extremes ranging from 15 to 79 years.

The age group between 25 and 34 years is predominant, with a rate of 28%, followed by patients aged 35–49 years with 26.9%.

Half of the population surveyed (57.4%) reported being married, with a significant difference between the two groups (*P*<0.036). The widowers seemed to present the most depressive episodes, 52.38% against 47.62%.

However, the exercise of professional activity was not related to the occurrence of depressive episodes in our population (*P*=0.627).

### Risk behaviors

The prevalence of depression appears to be higher in heavy smokers than in nonsmokers.

In our series, 54.3% of our patients who presented with a current depressive episode were smokers (*P*=000.1).

Exposure to tobacco in the workplace was usually found; 44.25% of our candidates reported a current depressive episode. However, exposure at home was found in 58.16% of cases (*P*=0.0001).

In the case of dual exposure (at home and work), 65.5% of our participants had a depressive episode.

**Figure 2:**
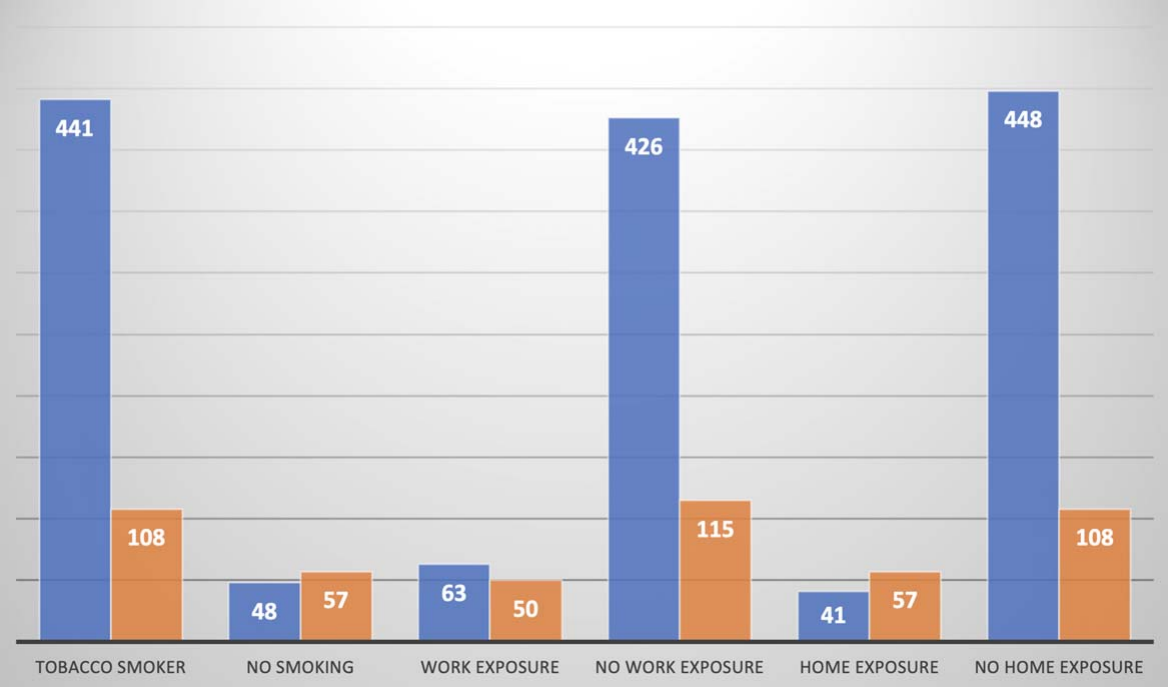
Depression and risk behaviors.

On the other hand, in the consumption of alcohol found in our population, depressive disorders were present at a rate of 71.43% in the habitual consumers of alcohol (*P*=000.1) (Fig. 2).

### Social relationships and depressive disorders

A better understanding of social contexts could help identify key components of social interactions that are more strongly associated with better mental health and reduced symptoms of anxiety and depression.

In our study, time spent with friends (duration >1.5 h) and the presence of children and spouses were evaluated as protective factors against depression.

On the other hand, less than half (35%) of older adults with depression reported spending no more than 1.5 h with friends (*P*=0.001).

In addition, the survey revealed that having a child was not statistically significant between the two groups (*P*=0.284).

However, adults with spouses were not likely to experience depressive episodes in our population (*P*=0.244).

Thus, individuals with all types of support (confidant, emotional, and instrumental) had a higher likelihood of consulting for their depressive symptoms than those with less support (Table 2).

**Table 2:** The social relationships and depressive disorders

	Current depressive episode		
	G1 (%)	G2 (%)	*P*
Time spent with friends (duration >1.5 h)			
Yes	183 (100)	0 (0)	0.001
No	306 (64.97)	165 (35.03)	
Spouse			
Yes	292 (76.44)	90 (23.56)	0.244
No	197 (72.43)	75 (27.57)	
Having children			
Yes	276 (73.21)	101 (26.8)	0.284
No	213 (76.9)	64 (23.1)	

### PA and depressive disorders

This study examined cross-sectional associations between PA, mental health, and anxiety-depressive symptoms in the population of Fez-Loulja.

In all, 5.58% of the physically active participants had a depressive episode, compared to 27.71% of the nonphysically active participants who had a depressive episode.

PA volume was positively associated with mental health and inversely associated with depressive symptoms (*P*=0.001).

After controlling for PA volume, active youth participating in team sports had better mental health than those participating in individual activity (Table 3).

**Table 3:** The relationship between physical activity and current depressive episode

	Current depressive episode		
	G1 (%)	G2 (%)	*P*
Physical activity			
Yes	69 (94.52)	4 (5.48)	0.001
No	420 (72.29)	161 (27.71)	

### Nutrition and depression

The dietary typologies identified according to the total score calculated for the two groups, G1 and G2, reveal that more than half (83.5%) of the study population on a MD did not experience a depressive episode, compared to only 16.4% of the participants who had a depressive episode.

Our investigation also shows that processed food is strongly related to the current depressive episode (*P*=0.0001).

Regarding the two remaining diets, the mixed diet, which is composed of both Mediterranean foods and processed foods, and the poor diet, represented by a low MDS (Mediterranean Diet Score) without processed products, the results were statistically significant between the two groups.

For the food typologies studied in our series, the difference is very significant between the two groups (Table 4).

**Table 4:** The relationship between diet and current depressive episodes in our population

	Current depressive episode		
Régime	G1 (%) – non-depressive	G2 (%) – depressive	*P*
Mediterranean diet			
Yes	223 (83.5)	44 (16.4)	0.0001
No	266 (68.7)	121 (31.3)	
Mixed diet			
Yes	147 (84)	28 (16)	0.001
No	342 (71.40)	137 (28.6)	
Poor diet			
Yes	36 (60)	24 (40)	0.006
No	453 (76.26)	141 (23.74)	
Processed diet			
Yes	83 (54.61)	69 (45.39)	0.0001
No	406 (80.88)	96 (19.12)	

### The multinomial probit model

The multinomial probit regression model was used to examine the factors that influence the occurrence of depressive disorder in our population in the Fez-Meknès region.

Table 5 represents the maximum likelihood estimates of the multinomial probit regression model. The table shows that the estimated log-likelihood is −256.05861. Since the maximum likelihood estimates are between 0 and 1, the log-likelihood estimate is always negative. The *χ* ^2^ distribution statistic was 154.99 (degree of freedom was 14).

**Table 5:** The probit regression model of the study

Variables	Coefficients	SE	*Z*	*P*>*χ* ^2^
Location	0.5424419	0.1457725	3.72	0.000
Gender	0.5427002	0.1930894	2.81	0.005
Have a spouse	−0.5829164	0.2081258	−2.80	0.005
Age	−0.0011001	0.0051337	−0.21	0.830
Having children	−0.6505328	0.2381116	−2.73	0.006
Having a professional activity	−0.3121814	0.1883984	−1.66	0.098
Smoking tobacco	1.030184	0.203922	5.05	0.000
Exposure to tobacco at work	−1.362973	0.3095416	−4.40	0.000
Exposure to tobacco at home	−0.4265365	0.3113836	−1.37	0.171
Double exposure to tobacco	0	–	–	–
Consumption of alcohol	0.6174803	0.3743833	1.65	0.099
Time spent with friends	−0.7060159	0.1330937	−5.30	0.000
Mediterranean diet	−1.196551	0.1822235	−6.57	0.000
Mixed diet	−0.9594507	0.1906727	−5.03	0.000
Poor diet	−0.2927799	0.2257399	−1.30	0.195
Processed diet	0	–	–	–
-constant	1.11779	0.8143056	1.37	2.7138

Log pseudolikelihood: −256.05861; number of observations: 654; *P*>*χ* ^2^: 0.0000; pseudo-*R* ^2^: 0.3068.

The positive sign of the coefficient of the variable means that by changing the variable by one unit, participants can be expected to be more likely to be in the top category. On the other hand, the negative sign of the coefficient of the variable means that by changing the variable by one unit, one can expect participants to be more likely to belong to the lower category.

The ordered probit regression revealed that being rural, a smoker, an alcohol user, and having no spouse significantly increased depression in participants.

The coefficient of age was negatively related to the probability of the occurrence of age-related depression; however, this factor was not significant in the model.

Thus, having a spouse and/or children and spending time with friends on a healthy diet significantly decreased depression in our population.

## Discussion

### Depression and PA

Depression is the biggest factor affecting patients’ quality of life^3^.

PA has been shown to improve patients’ clinical status in areas such as fatigue, depressive symptoms, sleep disturbance, and quality of life^11^.

One of the mechanisms involved in the effect of exercise and depression is endorphins.

Endogenous endorphins are opioid polypeptides secreted primarily by the hypothalamic–pituitary axis during intense exercise, excitement, and pain, and their actions resemble those of opiates in their ability to produce analgesia and a sense of well-being^12^.

The opioid system plays a key role in mediating analgesia and social attachment and may also affect depression, given the link between beta-endorphins and depressive symptoms^13^–^16^.

In addition, the mechanisms by which exercise may improve depression remain unclear, primarily due to the methodological limitations of existing research^17^.

Numerous studies have shown that men and women with obesity have a 55% increased risk of developing depression, while people with depression have a 58% increased risk of developing obesity^18^–^20^.

Specifically, aerobic exercise indicated a moderate clinical effect, while mixed and resistance exercise indicated large effect sizes. Furthermore, when compared to other established treatments (i.e. cognitive behavioral therapy and antidepressants), exercise appeared to produce the same results (Table 6).

**Table 6:** Data on depression and physical activity

Authors	*N*	Age	PA	Duration	Rhythm	Test	Results
Burini *et al*.^21^	26	62.7–65.7	Aerobic training	50 min	3 times/week for 7 weeks	BDI-II	No significant change
Lee *et al*.^22^	20	68.4–70.1	Dance exercise	30 min	5 times/week for 6 weeks	BDI-II	Positive effect on balance, activities of daily living, and depressive disorder status
Tanaka *et al*.^23^	20	64.6–64.8	Aerobic training	60 min	3 times/week for 6 weeks	HADS	No depressive symptoms (HADS=8 or higher)
Dereli and Yaliman^24^	32	61.3–66.5	Stretching, relaxation exercises	45 min	3 times/week for 10 weeks	BDI-II	Improvement in BDI and activities of daily living
Schmitz-Hübsch *et al*.^25^	56	63.8	Qi-Gong	60 min	8 weeks with a break of 8 weeks	MADRS	Depression and nonmotor symptoms decreased in the treatment group
Khallaf and Fathy^26^	30	49–70	Aerobic training	6–20 min	6 weeks	HDRS	More efficacy in activities of daily living and depressive symptoms
Cheon *et al*.^27^	23	62.3–65.6	Stretching, relaxation exercises	40–50 min	3 times/week for 8 weeks	BDI-II	No improvement in Parkinsonian symptoms and depression
Sajatovic *et al*.^28^	30	70	Self-guided exercises	40 min	3 times/week for 12 weeks	MADRS	Significant improvement in MADRS

BDI-II, Beck Depression Inventory; HADS, Hospital Anxiety and Depression Scale; MADRS, Montgomery-Asberg Depression Rating Scale; PA, physical activity.

The effectiveness of PA, such as general exercise and balance training, decreases depressive symptoms. However, stretching and Tai Chi do not affect depressive symptoms but can improve physical function and quality of life in patients^21^,^25^,^27^,^28^.

Indeed, published evidence shows that exercise and PA interventions are generally successful in reducing symptoms of depression^25^.

In a recent study, the authors were able to demonstrate that patients with major depression receiving aerobic training at home or in a supervised group setting achieved reductions in depression compared to standard antidepressant medications (sertraline) and greater reductions in depression compared to placebo controls^7^.

Numerous studies have shown that people with the major depressive disorder who follow an aerobic exercise program are equally likely to go into remission^23^,^26^ as those taking standard antidepressants (sertraline) or combined medication and exercise^21^.

PA is of increasing interest in the prevention of mental disorders in youth. Recent studies have shown a reduction in depressive symptoms with PA. Therefore, PA may be a potential target for the treatment and mitigation of negative symptoms such as depressive disorders^29^–^33^.

In our series, 94.52% of the participants who engaged in PA did not experience a depressive episode (*P*=0.001). Our results were consistent with the literature; therefore, PA interventions may be a therapeutic target when treating depression.

### Depression and nutrition

The multifactorial relationship between diet, mood disorders, and depression is bidirectional and complex^18^.

Based on the study by Gantenbein and Kanaka-Gantenbein^34^, it is established that a healthy diet, in terms of adherence to the MD, rich in fruits, vegetables, olive oil, herbs, and spices, and a high intake of fiber, can have a beneficial effect on mental health.

It has been described that the beneficial effects of the MD can be mainly attributed to its many components rich in anti-inflammatory and antioxidant properties^35^–^37^.

In addition, the MD may contribute to improved reproductive health, modify the risk of neurodegenerative diseases and protect against depression and psychosocial maladjustment. The beneficial effects of the MD can be enhanced by increased PA as part of a healthy and balanced lifestyle^34^.

Although complex, dietary interventions can include nutrient interventions (e.g. zinc, omega-3 fatty acids), food interventions (e.g. green tea, olive oil), and whole food interventions (e.g. MD)^18^,^38^,^39^.

The great variety and diversity of bioactive compounds present in the various dietary interventions, as well as the pleiotropic properties of these compounds, make their effects and the study of these effects inherently complex^19^.

This is further complicated by the lack of research that has studied the comparative effectiveness of a wide range of potentially therapeutic dietary interventions (e.g. MD, ketogenic diet, or caloric restriction), which differ considerably in their macronutrient and micronutrient composition (Table 7).

**Table 7:** The effect of diet intake on depression

Authors	Years	Country	Diet	Study	Results	*P*
Sánchez-Villegas *et al*.^40^	2009	Spain	M	C	Greater adherence to the Mediterranean diet is associated with a reduced risk of self-reported depression (traditional)	<0.001
Okubu *et al*.^41^	2011	Japan	J	C	No significant association	0.59
Mamplekou *et al*.^20^	2010	Mediterranean Islands	M	O	No significant association	NS
Akbaraly *et al*.^39^	2009	England	T	C	Increased consumption of processed foods is associated with an increased likelihood of depressive symptoms	0.001
Chatzi *et al*.^35^	2011	Greece	O	C	No significant association	0.70
Nanri *et al*.^36^	2010	Japan	J	O	Greater adherence to the Japanese diet is associated with a reduced risk of depressive symptoms	<0.001
Jacka *et al*.^37^	2011	Norway	N	O	Greater adherence to the Norwegian diet is associated with a reduced risk of depressive symptoms in men	0.02
Parker *et al*.^42^	2010	South Korea	L	CT	Greater adherence to a healthy diet is associated with a lower mean	<0.01
Aihara *et al*.^38^	2011	Japan	E	CT	Greater adherence to eating balanced meals is associated with a reduced likelihood of depressive symptoms	<0.05

Diet category: E, balanced diet; J, Japanese; L, low calorie; M, Mediterranean; N, Norwegian; O, Western diet; T, processed foods. Study category: C, cohort; CT, case–control; O, observational.

NS, not significant.

In this regard, many studies have concluded the relationship between increased potential diet and depression. Thus, significant differences have been recorded between diet and depression. The team of Sánchez-Villegas *et al*.^40^ demonstrated that adherence to MD ensures an adequate intake of B vitamins and W-3 fatty acids^42^. A protective role on depression has been suggested for these two nutrients. Our results indicate that the diets intervene directly on the psychological state of the participants (in our framework: the expression of symptoms related to depression).

As a result, 45.39% of the participants in our series were on a processed diet and presented with a depressive disorder (*P*=0.0001), which is in line with the results of the team of Akbaraly *et al*.^39^. The results suggest that a diet based on processed foods is a risk factor for depression.

However, 16.4% of participants on the MD had a depressive episode. Our data are consistent with the literature. This suggests that diet plays a central role in the body’s antioxidant metabolism by reducing biological stress. Finally, intervention in the dietary axis appears beneficial in reducing negative symptoms related to depression.

### Depression and social relationships

Social support may be particularly important in combating depression in systematically disadvantaged groups^43^. One reason given for this phenomenon is the strong social support provided by kinship networks.

Support from family and friends had protective effects on depression risk; however, after mutual adjustment, only family support remained statistically significant (Table 8).

**Table 8:** Data on depression and social support

Authors	Year	Country	Type of support	Study	Results	OR (IC 95%)
Almeida *et al*.^44^	2009	The United States	Friends	C	The effect of support from friends was protective against depression	0.76 (0.57–1.01)
Barger *et al*.^45^	2014	The United States	Friends	O	Loneliness and unmet support were associated with depressive disorders	0.59 (0.38–0.92)
Barth *et al*.^46^	2014	Switzerland	Friends	O	Reduced social support correlated with depression	0.53 (0.33–0.86)
Hefner and Eisenberg^47^	2009	The United States	Friends	M	A six-fold increase in the risk of depressive symptoms in students with poor-quality social support and those with good-quality social support	0.77 (0.39–1.52)
McKenzie *et al*.^48^	2013	The United States	Friends	C	The association between social contact and depression was stronger for men than for women	0.37 (0.22–0.63)
Stafford *et al*.^49^	2011	England	Friends	C	Negative, but not positive, exchanges with other family members and with friends were associated with depression	0.98 (0.91–1.05)
Wade and Kendler^50^	2000	The United States	Friends/family	O	The risk of major depression was inversely associated with supportive relationships with spouses and significant others and directly associated with problems in these relationships	0.96 (0.86–1.07

C, cohort; M, a meta-analysis; O, observational; OR, odds ratio.

At higher levels of family support, foreign-born Mexicans and African Americans had a lower risk of depression than at low levels of family support^44^.

The risk of major depression in the past year was inversely associated with supportive relationships with spouses and significant others and directly associated with problems in those relationships (e.g. too many demands, criticism, tension, and disagreements)^50^.

History of major depression in one twin significantly predicted low parental and spousal support, as well as problems with relatives and friends, in his co-twin. The relationship between social support and depression in women is even more complex^50^.

In addition, Hefner and Eisenberg^47^ showed that students with characteristics different from most other students, such as race or minority ethnicity, international status, and low socioeconomic status, were at greater risk of social isolation. For their part, students with lower quality social support, as measured by the multidimensional Perceived Social Support Scale, were more likely to experience mental health problems, including a six-fold increase in risk for depressive symptoms compared to students with high-quality social support. These findings may help healthcare administrators and providers more effectively identify the population of students at high risk for mental illness and develop effective interventions to address this important and growing public health problem^47^.

Compared with respondents who had 10 or more friends, the odds ratios (ORs) for depression were 4.01 (95% CI=1.89–8.50) and 1.86 (95% CI=0.92–3.79), respectively, for men and women who did not have close friends^48^.

According to Stafford *et al*., positive and negative exchanges with partners and children were independently associated with depression, controlling for age, gender, wealth, and initial depression. Negative, but not positive, exchanges with other family members and with friends were associated with depression. The association between depression and positive–negative exchanges was weaker for those over 70 than for those between 50 and 70^49^. Negative and positive exchanges with partners and children appear to be equally important in the development of depression, but negative exchanges with family and friends contribute to depression, while positive exchanges do not^49^.

Our study concluded that social contact (time spent with friends >1.5 h) remained strongly associated with reduced depressive symptoms when comparing the two groups (*P*=0.001). However, digital social networks are one of the fastest growing industries, creating a new platform for establishing social contacts at a distance. It is important to explore how to maximize the potential of digital social networks to strengthen social ties while balancing their negative effects.

### Limitations

The type, extent, and impact of measurement error, as well as interindividual variation, is an open area of research.

## Conclusion

Exercise and diet are nonpharmaceutical treatments of choice for depression. The benefits of exercise and a balanced diet may also persist beyond the end of treatment, unlike antidepressant medications.

However, a good social relationship seems to be a protective factor and has a prophylactic role.

Future studies need to test whether the brain regions identified in this review may be neurobiological markers of depression that could serve as targets for exercise-based treatments for depression.

## Ethical approval

Not applicable.

## Consent

Not applicable.

## Sources of funding

Not applicable.

## Conflicts of interest disclosure

The authors declare that they have no conflicts of interest.

## Research registration unique identifying number (UIN)

1. Name of the registry: not applicable.
2. Unique identifying number or registration ID: not applicable.
3. Hyperlink to your specific registration (must be publicly accessible and will be checked): not applicable.

## Provenance and peer review

Not commissioned, externally peer-reviewed.

## References

[1] Park LT Zarate CA . Depression in the primary care setting. N Engl J Med 2019;380:559–568. 30726688 10.1056/NEJMcp1712493 PMC6727965

[2] Hauenstein EJ . Depression in adolescence. J Obstet Gynecol Neonatal Nurs 2003;32:239–248. 10.1177/0884217503252133 12685676

[3] Rakel RE . Depression. Prim Care 1999;26:211–224. 10318745 10.1016/s0095-4543(08)70003-4

[4] Alexopoulos GS . Depression in the elderly. Lancet 2005;365:1961–1970. 15936426 10.1016/S0140-6736(05)66665-2

[5] Cuijpers P Quero S Dowrick C . Psychological treatment of depression in primary care: recent developments. Curr Psychiatry Rep 2019;21:129. 31760505 10.1007/s11920-019-1117-x PMC6875158

[6] Choi KW Kim YK Jeon HJ . Comorbid anxiety and depression: clinical and conceptual consideration and transdiagnostic treatment. Adv Exp Med Biol 2020;1191:219–235. 32002932 10.1007/978-981-32-9705-0_14

[7] Hao Y Ge H Sun M . Selecting an appropriate animal model of depression. Int J Mol Sci 2019;20:4827. 31569393 10.3390/ijms20194827 PMC6801385

[8] Pitsillou E Bresnehan SM Kagarakis EA . The cellular and molecular basis of major depressive disorder: towards a unified model for understanding clinical depression. Mol Biol Rep 2020;47:753–770. 31612411 10.1007/s11033-019-05129-3

[9] Yang L Zhao Y Wang Y . The effects of psychological stress on depression. Curr Neuropharmacol 2015;13:494–504. 26412069 10.2174/1570159X1304150831150507 PMC4790405

[10] Agha RA Sohrabi C Mathew G . The PROCESS 2020 guideline: updating consensus Preferred Reporting Of CasE Series in Surgery (PROCESS) guidelines. Int J Surg 2020;84:231–235. 33189880 10.1016/j.ijsu.2020.11.005

[11] Dinas PC Koutedakis Y Flouris AD . Effects of exercise and physical activity on depression. Ir J Med Sci 2011;180:319–325. 21076975 10.1007/s11845-010-0633-9

[12] Fichna J Janecka A Costentin J . The endomorphin system and its evolving neurophysiological role. Pharmacol Rev 2007;59:88–123. 17329549 10.1124/pr.59.1.3

[13] Zadina JE . Isolation and distribution of endomorphins in the central nervous system. Jpn J Pharmacol 2002;89:203–208. 12184722 10.1254/jjp.89.203

[14] Okada Y Tsuda Y Bryant SD . Endomorphins and related opioid peptides. Vitam Horm 2002;65:257–279. 12481550 10.1016/s0083-6729(02)65067-8

[15] Hebb ALO Poulin JF Roach SP . Cholecystokinin and endogenous opioid peptides: Interactive influence on pain, cognition, and emotion. Prog Neuropsychopharmacol Biol Psychiatry 2005;29:1225–1238. 16242828 10.1016/j.pnpbp.2005.08.008

[16] Terskiy A Wannemacher KM Yadav PN . Search of the human proteome for endomorphin-1 and endomorphin-2 precursor proteins. Life Sci 2007;81:1593–1601. 17964607 10.1016/j.lfs.2007.09.025 PMC2144908

[17] Mead GE Morley W Campbell P . Exercise for depression. Cochrane Database Syst Rev 2009;3:CD004366. 10.1002/14651858.CD004366.pub4 19588354

[18] Marx W Lane M Hockey M . Diet and depression: exploring the biological mechanisms of action. Mol Psychiatry 2021;26:134–150. 33144709 10.1038/s41380-020-00925-x

[19] Lassale C Batty GD Baghdadli A . Healthy dietary indices and risk of depressive outcomes: a systematic review and meta-analysis of observational studies. Mol Psychiatry 2019;24:965–986. 30254236 10.1038/s41380-018-0237-8 PMC6755986

[20] Mamplekou E Bountziouka V Psaltopoulou T . Urban environment, physical inactivity and unhealthy dietary habits correlate to depression among elderly living in eastern Mediterranean islands: the MEDIS (MEDiterranean ISlands Elderly) study. J Nutr Health Aging 2010;14:449–455. 20617287 10.1007/s12603-010-0091-0 PMC12880279

[21] Burini D Farabollini B Iacucci S . A randomised controlled cross-over trial of aerobic training versus Qigong in advanced Parkinson’s disease. Eura Medicophys 2006;42:231–238. 17039221

[22] Lee N-Y Lee D-K Song H-S . Effect of virtual reality dance exercise on the balance, activities of daily living, and depressive disorder status of Parkinson’s disease patients. J Phys Ther Sci 2015;27:145–147. 25642060 10.1589/jpts.27.145 PMC4305547

[23] Tanaka K de Quadros AC Santos RF . Benefits of physical exercise on executive functions in older people with Parkinson’s disease. Brain Cogn 2009;69:435–441. 19006643 10.1016/j.bandc.2008.09.008

[24] Dereli EE Yaliman A . Comparison of the effects of a physiotherapist-supervised exercise programme and a self-supervised exercise programme on quality of life in patients with Parkinson’s disease. Clin Rehabil 2010;24:352–362. 20360152 10.1177/0269215509358933

[25] Schmitz-Hübsch T Pyfer D Kielwein K . Qigong exercise for the symptoms of Parkinson’s disease: a randomized, controlled pilot study. Mov Disord 2006;21:543–548. 16229022 10.1002/mds.20705

[26] Khallaf M Fathy H . Effect of treadmill training on activities of daily living and depression in patients with Parkinson’s disease. Middle East Curr Psychiatry 2011;18:144–148.

[27] Cheon S-M Chae B-K Sung H-R . The efficacy of exercise programs for Parkinson’s disease: Tai Chi versus combined exercise. J Clin Neurol 2013;9:237–243. 24285965 10.3988/jcn.2013.9.4.237 PMC3840134

[28] Sajatovic M Ridgel AL Walter EM . A randomized trial of individual versus group-format exercise and self-management in individuals with Parkinson’s disease and comorbid depression. Patient Prefer Adherence 2017;11:965–973. 28579759 10.2147/PPA.S135551 PMC5449131

[29] Carek PJ Laibstain SE Carek SM . Exercise for the treatment of depression and anxiety. Int J Psychiatry Med 2011;41:15–28. 21495519 10.2190/PM.41.1.c

[30] Ströhle A . Physical activity, exercise, depression and anxiety disorders. J Neural Transm 2009;116:777–784. 18726137 10.1007/s00702-008-0092-x

[31] Arida RM Cavalheiro EA Scorza FA . From depressive symptoms to depression in people with epilepsy: contribution of physical exercise to improve this picture. Epilepsy Res 2012;99:1–13. 22055354 10.1016/j.eplepsyres.2011.10.012

[32] Cena H Vandoni M Magenes VC . Benefits of exercise in multidisciplinary treatment of binge eating disorder in adolescents with obesity. Int J Environ Res Public Health 2022;19:8300. 35886152 10.3390/ijerph19148300 PMC9315465

[33] Gray ZJ Shields GS Sichko S . Neural and peripheral markers of reward during positive social evaluation are associated with less clinician-rated depression symptom severity in adolescence. Compr Psychoneuroendocrinol 2022;11:100149. 35856064 10.1016/j.cpnec.2022.100149 PMC9287766

[34] Gantenbein KV Kanaka-Gantenbein C . Mediterranean diet as an antioxidant: the impact on metabolic health and overall wellbeing. Nutrients 2021;13:1951. 34204057 10.3390/nu13061951 PMC8227318

[35] Chatzi L Melaki V Sarri K . Dietary patterns during pregnancy and the risk of postpartum depression: the mother-child ‘Rhea’ cohort in Crete, Greece. Public Health Nutr 2011;14:1663–1670. 21477412 10.1017/S1368980010003629

[36] Nanri A Kimura Y Matsushita Y . Dietary patterns and depressive symptoms among Japanese men and women. Eur J Clin Nutr 2010;64:832–839. 20485303 10.1038/ejcn.2010.86

[37] Jacka FN Mykletun A Berk M . The association between habitual diet quality and the common mental disorders in community-dwelling adults: the Hordaland Health study. Psychosom Med 2011;73:483–490. 21715296 10.1097/PSY.0b013e318222831a

[38] Aihara Y Minai J Aoyama A . Depressive symptoms and past lifestyle among Japanese elderly people. Community Ment Health J 2011;47:186–193. 20455023 10.1007/s10597-010-9317-1

[39] Akbaraly TN Brunner EJ Ferrie JE . Dietary pattern and depressive symptoms in middle age. Br J Psychiatry 2009;195:408–413. 19880930 10.1192/bjp.bp.108.058925 PMC2801825

[40] Sánchez-Villegas A Henríquez P Bes-Rastrollo M . Mediterranean diet and depression. Public Health Nutr 2006;9:1104–1109. 17378948 10.1017/S1368980007668578

[41] Okubo H Miyake Y Sasaki S . Dietary patterns during pregnancy and the risk of postpartum depression in Japan: the Osaka Maternal and Child Health Study. Br J Nutr 2011;105:1251–1257. 21144112 10.1017/S0007114510004782

[42] Parker G Gibson NA Brotchie H . Omega-3 fatty acids and mood disorders. Am J Psychiatry 2006;163:969–978. 16741195 10.1176/ajp.2006.163.6.969

[43] Mechakra-Tahiri S-D , Relations sociales et troubles dépressifs chez les personnes âgées au Québec: Interactions avec le genre et la région de résidence [Social relations and depressive disorders among the elderly in Quebec: interactions with gender and region of residence]. ProQuest Diss Theses, 2008, 222. https://search.proquest.com/dissertations-theses/relations-sociales-et-troubles-dépressifs-chez/docview/304801100/se-2?accountid=13042%0Ahttp://oxfordsfx.hosted.exlibrisgroup.com/oxford?url_ver=Z39.88-2004&rft_val_fmt=info:ofi/fmt:kev:mtx:dissertation&gen.

[44] Almeida J Subramanian SV Kawachi I . Is blood thicker than water? Social support, depression and the modifying role of ethnicity/nativity status. J Epidemiol Community Health 2011;65:51–56. 19910646 10.1136/jech.2009.092213

[45] Barger SD Messerli-Bürgy N Barth J . Social relationship correlates of major depressive disorder and depressive symptoms in Switzerland: nationally representative cross sectional study. BMC Public Health 2014;14:273. 24656048 10.1186/1471-2458-14-273 PMC3994328

[46] Barth J Hofmann K Schori D . Depression in early adulthood: prevalence and psychosocial correlates among young Swiss men. Swiss Med Wkly 2014;144:w13945. 24723252 10.4414/smw.2014.13945

[47] Hefner J Eisenberg D . Social support and mental health among college students. Am J Orthopsychiatry 2009;79:491–499. 20099940 10.1037/a0016918

[48] McKenzie LE Polur RN Wesley C . Social contacts and depression in middle and advanced adulthood: findings from a US national survey, 2005–2008. Int J Soc Psychiatry 2013;59:627–635. 24146434 10.1177/0020764012463302

[49] Stafford M McMunn A Zaninotto P . Positive and negative exchanges in social relationships as predictors of depression: evidence from the English Longitudinal Study of Aging. J Aging Health 2011;23:607–628. 21220351 10.1177/0898264310392992

[50] Wade TD Kendler KS . The relationship between social support and major depression: cross-sectional, longitudinal, and genetic perspectives. J Nerv Ment Dis 2000;188:251–258. 10830561 10.1097/00005053-200005000-00001

